# Liver Segment Disposition of Hepatocellular Carcinoma Predicts Microvascular Invasion

**DOI:** 10.1155/2023/5727701

**Published:** 2023-05-31

**Authors:** Arnold Nongmoh Forlemu, Raissa Nana Sede Mbakop, Praneeth Bandaru, Vijay Gayam, Hamsika Moparty, Tomoki Sempokuya, Faruq Pradhan, Madhavi Reddy, Marco Olivera

**Affiliations:** ^1^Department of Gastroenterology and Hepatology, The Brooklyn Hospital Center, Brooklyn, NY, USA; ^2^Department of Internal Medicine, Piedmont Athens Regional, Athens, GA, USA; ^3^Department of Internal Medicine, The Brooklyn Hospital Center, Brooklyn, NY, USA; ^4^Department of Gastroenterology and Hepatology, University of Nebraska Medical Center, Omaha, NE, USA

## Abstract

**Background:**

Hepatocellular carcinoma (HCC) is a leading cause of cancer morbidity and mortality. Findings of microvascular invasion (MVI) in patients with HCC have emerged as an important prognostic factor for poor survival after tumor resection.

**Aim:**

This study evaluated the relation between MVI and HCC within various anatomical Couinaud's segments of the liver.

**Method:**

A multicenter retrospective review of HCC records was conducted from 2012 to 2017. HCC cases were identified using ICD-9 and 10 codes 155, C22.0, and C22.8. HCC patients who underwent liver transplants were included in this study. Liver segment of the location of HCC was obtained from radiographic records, and MVI information was obtained from pathology reports. Segmental distributions of HCC in MVI versus non-MVI groups were compared using Wilcoxon rank sum tests. *p* value was set at <0.05.

**Results:**

We analyzed 120 HCC patients who underwent liver transplantation. The mean age of our cohort was 57 years, and the most common etiology of liver disease was hepatitis C at 58.3%. The median HCC size was 3.1 cm, and MVI was present in 23.3% of the explanted specimens. MVI was 2 to 3 times significantly higher in patients with HCC affecting segments 2 and 3 and segments 4b and 5 (*p* = 0.01). Moreover, median survival was significantly lower in patients with MVI versus those without MVI (50 vs. 137 months, *p* < 0.05).

**Conclusion:**

MVI was significantly higher in HCC tumors located in liver segments 2 and 3 and 4b and 5, and survival was lower in patients with MVI compared with those without.

## 1. Introduction

Primary liver cancer, the majority of which is hepatocellular carcinoma (HCC), is the sixth most common cancer worldwide and the third leading cause of cancer-related deaths [1]. By 2025, it is estimated that over one million individuals will be affected by liver cancer annually [2]. In the United States, liver cancer incidence rates have more than tripled since 1980, with death rates doubling during this time, associated with a significant socioeconomic burden [3]. Advances in imaging procedures and close monitoring have led to an increase in early detection and better treatment outcomes [4, 5]. However, despite potential curative therapy with surgical resection, liver transplant, and locoregional therapies, HCC recurrence remains elevated at 70% within 5 years [6].

Microvascular invasion (MVI), defined by the presence of tumor cells in portal vein branches and tracts, central veins in noncancerous liver tissue, in large capsule vessels, noncapsular fibrous septa, or in a vascular space lined by endothelial cells [7], has emerged as one of the most important risk factors for tumor recurrence and is a prognostic factor for poor survival following tumor resection and liver transplantation [8–10]. It has also been suggested that predicting MVI may also affect locoregional treatments and chemotherapy protocols [11]. Unlike macrovascular invasion, MVI is a histological and not a radiological diagnosis [12, 13]. Characterizing MVI for optimal HCC management is critical, given the difficulty of identifying MVI prior to therapy and its strong impact on clinical outcomes. Biochemical and imaging findings that have been used to suggest MVI include des-gamma-carboxy prothrombin (DCP) and alpha-fetoprotein (AFP) levels, disruption of the capsule, irregular tumor margin, peritumoral enhancement, multifocal tumor, increased tumor size, and increased glucose metabolism on positron emission tomography-computed tomography [11, 14–17]. On the other hand, Jakhete et al., in 2016, found that Milan criteria, AFP, tumor differentiation, and multilobar involvement were not predictive of MVI [18].

Rarely have studies investigated the Couinaud segmental distribution of HCC as a potential predictor of MVI [11]. Liver segments as defined by Couinaud are subdivisions of the liver into eight functional segments with each segment having its own biliary drainage and vascular inflow and outflow [19]. The segments are numbered in a clockwise pattern with segment 1 corresponding to the caudate lobe, segments 2-4 comprising the functional left lobe, and segments 5-8 corresponding to the functional right lobe. A retrospective study from 2012 to 2017, conducted by Al-Azzawi et al. [20], on 98 HCC patients at the University of Massachusetts, found liver segment 8 to correlate the most with MVI [20].

Understanding the patterns and anatomical/segmental preferences of HCC and MVI within the liver could provide relevant observational and management information. In our study, we aim to identify whether there is a correlation between HCC's various anatomical liver segments and MVI in patients who have undergone liver transplantation.

## 2. Materials and Methods

### 2.1. Study Design

We conducted a multicenter retrospective review of records of HCC patients 18 years and above at the University of Nebraska Medical Center and The Brooklyn Hospital Center from 2010 to 2017. HCC cases were identified using the international classification of diseases, ninth and tenth revisions, and clinical modification (ICD-9 and 10 codes 155, C22.0, C22.8). All HCC cases were radiographically or biopsy confirmed. Patients had undergone liver transplants, with or without prior locoregional therapy for HCC treatment. Bland vs. tumor thrombus was differentiated using A-VENA criteria; the presence of ≥3 of the following for tumor thrombus: AFP > 1000 ng/dl, venous expansion, thrombus enhancement (difference in the Hounsfield units >20), neovascularity, and adjacent or continuity of HCC lesion with thrombus [21]. Tumor stage was determined according to the Barcelona Clinic Liver Cancer (BCLC) staging system [22]: size and number of viable tumors, number of nodules, portal vein invasion, and metastasis. Tumor cell differentiation was determined by the modified Edmondson-Steiner's classification [23]: grade 1, well differentiated; grade 2, moderately differentiated; and grade 3, poorly differentiated.

Liver segment location of the HCC was obtained from radiographic records. MVI status, size of vessels invaded, and satellite nodule information were obtained from histological pathology reports. Two hundred and fifty patients with HCC were identified for this study. Patients with preoperative radiologically diagnosed tumor thrombus, patients with a model for end-stage liver disease (MELD) score > 30, and patients outside of Milan criteria were excluded from this study (102 patients). Additionally, another 28 patients intraoperatively diagnosed with macrovascular invasion (defined as tumor tissue found in the portal vein, bile duct, or hepatic vein) were excluded.

Data on demographics, clinical history, cirrhosis etiologies, family history of cancer, and treatment options including posttransplant immunosuppression medications were obtained through chart review of electronic medical records. All research was conducted in accordance with both the Declarations of Helsinki and Istanbul, and the study was approved by the institutional review board with institutional review board (IRB) number 0770-21-EP. Informed consent was waived for this study.

### 2.2. Analytical Methods

Descriptive statistics for continuous data are given as mean ± standard deviation for baseline characteristics and as medians and interquartile ranges (IQRs, representing the range of the middle 50% of the data) for MVI statistics. Associations between categorical variables were assessed using Chi-square tests or Fisher's exact test when expected cell counts were low. Wilcoxon rank sum tests were used to examine differences in distributions of variables of interest between MVI and non-MVI groups. Statistical significance was set at a *p* value <0.05. All analyses were performed using SAS software version 9.4 (SAS Institute Inc., Cary, NC).

## 3. Results

One hundred and twenty patients with HCC diagnosis without portal vein thrombosis (PVT) who underwent liver transplantation between 2010 and 2017 were included in this study. Baseline characteristics of the patients are shown in Table 1. The mean age of the cohort was 57.5 ± 8.2 years, with males representing 74% and 83% of the individuals in our cohort being Caucasian. The most common etiology of cirrhosis was chronic hepatitis C virus infection, followed by alcohol abuse and nonalcoholic steatohepatitis (NASH) at 58.3%, 12.5%, and 9.2%, respectively. The median size of HCC was 3.1 cm. MVI was present in 28 explanted specimens (23.3%). Postoperative recurrence of HCC within the 7-year period of the study was 14% (Table 1).

**Figure 1:**
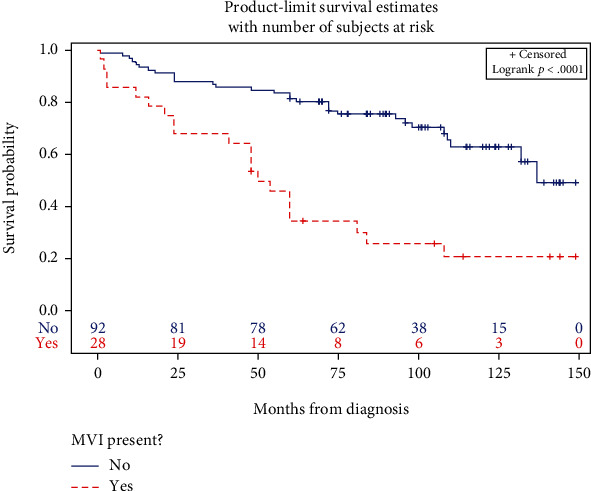
Survival curve by microvascular invasion. Blue line represents patients with HCC without MVI. Red line represents patients with HCC and MVI. Survival in red line category is significantly lower and decreases overtime compared to the blue line. MVI: microvascular invasion.

We studied the association between several parameters based on the presence or absence of MVI (Table 2). As expected, elevated AFP, advanced BCLC stage, HCC recurrence, and pathology staging had significantly higher proportions of MVI in the explanted specimen (*p* < 0.05). Patients with MVI had a higher median number of HCC-affected segments compared to those without MVI (2.0 vs. 1.0, *p* = 0.01). Interestingly, there was a trend of patients with hepatitis B infection (HBV) and HCC having a higher proportion of MVI compared to other non-HBV HCC etiologies; however, this trend was not statistically significant. Furthermore, MVI was 2 to 3 times more likely to be present in patients with HCC, affecting segments 2 and 3 and segments 4b and 5 compared to other segments (*p* = 0.01). Also, segments 5 and 8 showed a similar trend of higher presence of MVI; however, this trend was not statistically significant. Median survival was significantly lower in patients who had MVI (50 vs 137 months, *p* < 0.05) compared to those without MVI (Figure 1).

## 4. Discussion

Liver cancer, mostly hepatocellular carcinoma (HCC), is a major cause of cancer-related death, second only to lung cancer [1]. The incidence of liver cancer has continued to rise in the United States since the 1980s, and despite several available treatment options including liver transplant, surgery, and locoregional therapies, the 5-year recurrence of HCC has remained elevated [3, 6]. Management options for HCC often depend on tumor size, number, presence of vascular involvement, metastases, and functional status of the patient [20, 24]. Microvascular invasion (MVI) has emerged as a principal risk factor for tumor recurrence as well as survival posttumor resection or transplantation [8–10]. Likewise, MVI has been suggested to affect locoregional and chemotherapy protocols [11]. The prevalence of MVI in our study was 23%. Rodríguez-Perálvarez et al., in their 2013 systematic review, found the prevalence of MVI to vary widely between 15 and 57% [12]. The variation in prevalence was explained by geographic variations and lack of consensus on the definition of MVI. The etiology of cirrhosis might also have been responsible for this variation in prevalence. It is likely that the true prevalence lies somewhere in between those percentages like we found in our study. MVI, contrary to macrovascular invasion, is difficult to detect preoperatively. Biochemical and imaging findings, including des-gamma-carboxy prothrombin (DCP), alpha fetoprotein (AFP) levels, disruption of the capsule, irregular tumor margin, peritumoral enhancement, multifocal tumor, increased tumor size, tumor differentiation, multilobar involvement, increased glucose metabolism on positron emission tomography-computed tomography, and Milan criteria, have either been variable or controversial [11, 14–18].

Like with our study, Rodríguez-Perálvarez et al. (2013), Okamura et al. [14], and Sakata et al. [16] found tumor size, multifocal segment involvement, and AFP to be associated with MVI [12, 14, 16]. On the other hand, Jakhete et al. [18] did not find an association between MVI and multilobar HCC involvement, AFP level, and tumor differentiation [18]. This discrepancy could be explained by the fact that about half of their patients were outside Milan criteria, and the presence of PVT likely influenced the results of their study. As for the etiology of liver disease, only hepatitis B patients with HCC showed trends of a higher proportion of MVI in our study. It is possible that the aggressive nature of chronic hepatitis B infection that is known to accelerate the process of disease evolution from inflammation to tumorigenesis through various cancer-promoting mechanisms may be responsible for this trend [25]. Similarly, Huang et al., in 2017, found a history of hepatitis B infection to be associated with MVI, and like our study, this association was not statistically significant [26]. Conversely, Al-Azzawi et al. [20] did not find an association between the etiology of liver disease and MVI [20]. However, they did not include hepatitis B infection as an etiology of liver disease when correlating MVI and the etiology of liver disease. More attention should be granted to the association between HBV chronically infected patients and MVI in longitudinal studies.

Given the prime predictive and prognostic value of MVI in both recurrence and outcome of HCC, we investigated the association between the Couinaud segments of HCC, tumor localization, and the presence of MVI. On average, more than one segment was affected with HCC in our cohort. Segments 7 and 8 were more likely to be involved with HCC compared to other segments. Individually, HCC lesions located in segments 4, 5, and 8 had a higher proportion of MVI compared to other segments, although this was not statistically significant. Interestingly, when we analyzed HCC lesions found in more than one segment, lesions in both segments 2 and 3 and segments 4b and 5 had a 2 to 3 times significantly higher proportion of MVI compared to other combinations, 71.4 vs. 20.4 and 37.5 vs. 16.3, respectively. Lesions located in segments 4, 5, and 8 have traditionally been known as central HCCs [27]. These HCC locations adjacent to the main hepatic artery vascular structure have a dual blood supply from both left and right hepatic arteries, increasing their risk of MVI [28]. Segments 2 and 3, also known collectively as the left lateral segment of the liver and the topographic left lobe, have also been shown to sometimes receive blood from the hepatic artery and the left portal vein [29, 30]. This might explain why HCC lesions in both segments carried a higher proportion of MVI in our study. Our findings are like those of Al-Azzawi et al., who found a higher risk for MVI for HCCs located in segments 2, 4, and 8, although only that of segment 8 rose to statistical significance [20]. A limitation of our study is that differences in tumor biology between segments were not compared, and hence, more aggressive lesions in the above segments could affect our findings.

As mentioned earlier, MVI is one of the most important risk factors for tumor recurrence and a prognostic factor for survival post HCC resection or liver transplantation. Patients with MVI in our study had a tumor recurrence five times higher than that of those without MVI. Likewise, MVI patients had a median survival three times lower compared to those without MVI. Our findings are in line with other studies in the literature [8–10]. MVI has been used to discriminate clinical outcomes in patients with HCC within the Milan criteria, and it is suggested that it might be helpful in prognosis stratification in patients beyond this classification [31]. MVI may also act as a distinct tumor behavior marker and should be actively looked for and documented after tumor resection or transplantation [32]. To avoid underestimation, a thorough examination of the surgical specimen is mandatory and considered the standard way of confirmation. Accurate information obtained on the presence of MVI would likely play a role in refining the current HCC staging system and management options [32].

## 5. Conclusion

Our data indicate that the proportion of MVI is highest in HCC tumors located in the Couinaud segments 2 and 3 and 4b and 5. In addition, hepatitis B infection showed a higher trend of MVI compared to other etiologies. The number of HCC-affected segments had a higher association with MVI. MVI was higher in patients with HCC tumor recurrence and was associated with a lower survival post liver transplantation. We recommend a larger and prospective study to investigate the Couinaud segmental association with MVI and whether the involvement of HCC in these segments could be incorporated in preoperative therapeutic HCC management strategies.

## Figures and Tables

**Table 1:** Baseline characteristics.

Characteristics	Mean ± SD or *N* = 120, (%)
Age at diagnosis, years	57.5 ± 8.2
Gender	
Male	89 (74.2)
Female	31 (25.8)
Race	
White	100 (83.3)
Black	6 (5.0)
Asian	8 (6.7)
Other	6 (5.0)
Ethnicity	
Hispanic	5 (4.2)
Non-Hispanic	115 (95.8)
Etiology	
HCV	70 (58.3)
Alcohol	15 (12.5)
NAFLD	11 (9.2)
HBV	5 (4.2)
Alpha 1 anti-trypsin deficiency	4 (3.3)
Autoimmune conditions (^∗^)	10 (8.3)
Cryptogenic	5 (4.2)
Mean number of HCCs	1.5 ± 0.8
Mean number of segments affected by HCC	1.8 ± 0.9
Segments	
Single segment involved	60 (50.0)
Multi segments	60 (50.0)
Single lesion	81 (67.5)
Multifocal lesions	39 (32.5)
Single lesion/single segment	48 (59.3)
Single lesion/multi segments	33 (40.7)
Segment 1	1 (0.8)
Segment 2	17 (14.2)
Segment 3	19 (15.8)
Segment 4a	19 (15.8)
Segment 4b	14 (11.7)
Segment 5	29 (24.2)
Segment 6	39 (32.5)
Segment 7	42 (35.0)
Segment 8	43 (35.8)
HCC size, cm	3.1 ± 1.8
AFP	156.0 ± 507.4
Survival time, months	80.5 ± 40.7
Microvascular invasion	28 (23.3)
Recurrence	14 (14.0)

(^∗^) Autoimmune conditions: autoimmune hepatitis, primary biliary cholangitis, primary sclerosing cholangitis. HCC: hepatocellular carcinoma; AFP: alpha fetoprotein; HCV: hepatitis C virus; HBV: hepatitis B virus; NAFLD: nonalcoholic fatty liver disease; SD: standard deviation; *N*: number of cases.

**Table 2:** Findings by microvascular invasion.

Characteristics	Microvascular invasion	*p*-value
Median number of affected segments	2.0 vs. 1.0	0.01^∗^
Median number of HCCs	1.0 vs. 1.0	0.23
Median HCC tumor size	3.5 vs. 2.5	0.09
Median AFP	18.8 vs. 8.8	0.02^∗^
HCC etiology, %		
HCV	22.0 vs. 26.3	0.60
HBV	60.0 vs. 21.7	0.08
Alcohol	13.3 vs. 24.8	0.52
NAFLD	36.4 vs. 22.0	0.28
Segment		
Segment 1	0.0 vs. 23.5	1.00
Segment 2	35.3 vs. 21.4	0.22
Segment 3	26.3 vs. 22.8	0.77
Segment 4a	36.8 vs. 20.8	0.14
Segment 4b	35.7 vs. 21.7	0.31
Segment 5	34.5 vs. 19.8	0.10
Segment 6	25.6 vs. 22.2	0.68
Segment 7	23.8 vs. 23.1	0.93
Segment 8	25.6 vs. 22.1	0.66
Segment 2 and 3	71.4 vs. 20.4	0.01^∗^
Segment 4a and b	34.6 vs. 20.2	0.12
Segment 5 and 6	26.9 vs. 20.6	0.42
Segment 6 and 7	24.2 vs. 22.4	0.82
Segment 7 and 8	24.2 vs. 22.2	0.79
Segment 4b and 5	37.5 vs. 16.3	0.01^∗^
Segment 4a and 8	27.3 vs. 20.0	0.35
Segment 4b and 8	29.1 vs. 18.5	0.17
Segment 5 and 8	42.9 vs. 20.8	0.09
BCLC A, B, and C stages	18.5 vs. 36.0 vs. 66.7	0.04^∗^
HCC pathology stage, 1, 2, and 3	12.4 vs. 51.6 vs. 50.0	≤0.01^∗∗^
HCC recurrence	78.6 vs. 14.0	≤0.01^∗∗^
Median survival, months	50 vs. 137	0.01^∗^

HCC: hepatocellular carcinoma, AFP: alpha fetoprotein, HCV: hepatitis C virus, HBV: hepatitis B virus, NAFLD: Nonalcoholic fatty liver disease, BCLC: Barcelona Clinic Liver Cancer, ^∗^*p* < 0.05, ^∗∗^*p* < 0.01.

## Data Availability

Data would be made available upon reasonable request from the corresponding author.
